# Varicella Zoster Virus disrupts MAIT cell polyfunctional effector responses

**DOI:** 10.1371/journal.ppat.1012372

**Published:** 2024-08-07

**Authors:** Shivam. K. Purohit, Lauren Stern, Alexandra J. Corbett, Jeffrey Y. W. Mak, David P. Fairlie, Barry Slobedman, Allison Abendroth

**Affiliations:** 1 Infection, Immunity and Inflammation, School of Medical Sciences, Faculty of Medicine and Health, Charles Perkins Centre, University of Sydney, Sydney, New South Wales, Australia; 2 Department of Microbiology and Immunology, The University of Melbourne, at the Peter Doherty Institute for Infection and Immunity, Melbourne, Victoria, Australia; 3 ARC Centre of Excellence for Innovations in Peptide and Protein Science, Institute for Molecular Bioscience, University of Queensland, Brisbane, Queensland, Australia; Emory and Stanford Universities, UNITED STATES OF AMERICA

## Abstract

Mucosal-associated invariant T (MAIT) cells are unconventional T cells that respond to riboflavin biosynthesis and cytokines through TCR-dependent and -independent pathways, respectively. MAIT cell activation plays an immunoprotective role against several pathogens, however the functional capacity of MAIT cells following direct infection or exposure to infectious agents remains poorly defined. We investigated the impact of Varicella Zoster Virus (VZV) on blood-derived MAIT cells and report virus-mediated impairment of activation, cytokine production, and altered transcription factor expression by VZV infected (antigen+) and VZV exposed (antigen-) MAIT cells in response to TCR-dependent and -independent stimulation. Furthermore, we reveal that suppression of VZV exposed (antigen-) MAIT cells is not mediated by a soluble factor from neighbouring VZV infected (antigen+) MAIT cells. Finally, we demonstrate that VZV impairs the cytolytic potential of MAIT cells in response to riboflavin synthesising bacteria. In summary, we report a virus-mediated immune-evasion strategy that disarms MAIT cell responses.

## Author summary

Mucosal-associated invariant T (MAIT) cells are a uniquely specialised and substantial innate-T cell population that can rapidly respond to diverse bacterial and fungal pathogens through T cell receptor dependent recognition of riboflavin synthesis derived metabolite antigens. Additionally, MAIT cells can be triggered by local pro-inflammatory cues such as cytokines; therefore extending their functionality to non-riboflavin pathogens such as viral infections. Despite the capacity of MAIT cells to play a protective role against several classes of pathogens, there remains a dearth of studies investigating direct pathogenic suppression of MAIT cell functionality. Here, we investigate a previously uncharacterised interplay between MAIT cells and the causative agent of varicella (chickenpox) and shingles (zoster): Varicella Zoster Virus (VZV). VZV successfully infects and establishes lifelong latency within the host; in part to their ability to effectively manipulate several innate and adaptive axes of the host immune response. In this study, we report that VZV profoundly impairs MAIT cell activation in response to both riboflavin synthesis and cytokine stimulation, therefore resulting in a downstream paralysis of several effector functions such as cytokine production and cytotoxic potential. This work highlights a previously uncharacterised strategy of viral pathogens to effectively target and restrict the MAIT cell effector response.

## Introduction

Varicella Zoster Virus (VZV) is a highly successful human pathogen that causes varicella during primary infection and can later reactivate from latency and result in herpes zoster (HZ). A defining characteristic of VZV pathogenesis is the virus’ ability to encode several immune evasion strategies that impair pathogen detection systems whilst also infecting and disabling host effector immune cell populations. In particular, VZV evades classical T cell and Natural killer (NK) cell responses by downregulating surface expression of Major Histocompatibility Complex class-I [1], class II (MHC-II) [2], and NK cell activating ligands [3]. Furthermore, VZV productively infects and functionally disrupts several immune cell subsets such as monocytes [4], dendritic cells (DCs) [5,6], conventional T cells [7,8], and NK cells [9,10] enabling host wide hematogenous dissemination. A common feature of varicella infection is viremia accompanied with high viral load at respiratory mucosa and skin sites [11,12]. Clinically, primary varicella infection presents as cutaneous vesicular lesions which disrupt the normal skin architecture at these sites, and permit translocation of commensal microbes. Unsurprisingly, bacterial superinfections ranging from bacterial cellulitis to pneumonia and/or sepsis are a common complication arising from severe VZV infection [13–15].

Importantly, these barrier locations are also enriched with resident Mucosal Associated Invariant T (MAIT) cells [16,17] which typically express a semi-invariant T cell receptor (TCR) [18]. MAIT cells are the largest innate-adaptive immune cell population within the body and are exquisitely tuned to rapidly respond to deeply conserved microbial metabolic patterns expressed by commensal and pathogenic species. Specifically, the MAIT TCR binds to unstable microbial metabolite antigens such as 5-(2-oxopropylideneamino)-6-d-ribitylaminouracil (5-OP-RU) derived from the Vitamin B2 (riboflavin) biosynthesis pathway presented by the MHC-I related molecule MR1 [19,20]. Riboflavin biosynthesis is a metabolic process that is broadly conserved across diverse bacterial and fungal species [21]; however is not present within viral or mammalian systems.

TCR dependent stimulation of MAIT cells drives co-expression of transcription factors: RAR-related orphan receptor γ T (RORγt) and T box 21 (T-bet) [22], leading to the expression of several cytokines such as Interferon (IFN)-γ, Tumour necrosis factor (TNF), interleukin (IL)-17 and IL-22, as well as cytolytic capacity denoted by granzyme B and perforin expression [22–24]. Consequently, the cognate MAIT TCR-MR1 interaction enables MAIT cells to enact rapid polyfunctional responses against both commensal and pathogenic riboflavin synthesising organisms at several key mucosal barrier sites [25–27].

Under homeostatic conditions, the constant and steady diffusion of riboflavin metabolites from commensals across mucosal interfaces drives a T cell effector type-17 (Tc-17) like barrier maintenance and tissue repair signature [17,28–30]. However, during an infection setting, an intact riboflavin synthesising pathogen would also trigger several toll-like receptors (TLRs) within the antigen presenting cell (APC), resulting in a concomitant delivery of MR1 antigen presentation and innate signals to MAIT cells [16,31]. Indeed, MAIT cells are highly responsive to interleukin IL-18 and IL-12 signalling, which in combination with TCR stimulation drives a robust and prolonged functional response as well as proliferation [32].

Importantly, MAIT cells can be activated by cytokines such as IL-12 and IL-18 in the absence of TCR stimulation [33], extending the influence of MAIT cell functionality to viral and autoimmune diseases. Cytokine driven activation of MAIT cells drives a predominantly T-bet mediated response characterised by IFN-γ and granzyme B expression [22]. This robust anti-viral phenotype can result in protective efficacy against several viral infections such as Human Immunodeficiency Virus (HIV) [34], Hepatitis B Virus (HBV) [35], and Influenza [36].

Despite the ability of MAIT cells to rapidly respond to, and coordinate immune responses to, a vast array of pathogens, pathogen encoded strategies that directly impair MAIT cell effector functionality remain understudied. Our previous studies have revealed both modulation of MR1 antigen presentation [37], and direct infection of MAIT cells [38], therefore suggesting a direct targeting of the host MR1-MAIT cell axis by VZV for pathogenic gain. Therefore, this current study aimed to interrogate the direct impacts on MAIT cell functionality following VZV infection. Herein, we demonstrate a profound inhibition of MAIT cell response to both TCR-dependent and -independent activation, as shown by a significant abrogation of activation and cytokine expression. Strikingly, the suppression of effector response was observed in both VZV antigen positive as well as antigen negative MAIT cells. Finally, we reveal an impairment of cytotoxic capacity towards intact bacteria in VZV co-cultured MAIT cells. Overall, our report demonstrates a direct impact of viral infection on MAIT cell effector functionality following viral interaction and infection.

## Results

### VZV impairs MAIT cell activation

We sought to assess the direct outcome of VZV infection on MAIT cell functionality. Using a cell-associated infection model that closely replicates *in vivo* transmission of virus [38], we co-cultured VZV infected epithelial cells with human peripheral blood mononuclear cells (PBMCs). Following 24 hours of co-culture, we assessed MAIT cell responses to four distinct treatment conditions: 1. 5-OP-RU (TCR ligand treatment), 2. IL-12/IL-18 (cytokine treatment), 3. 5-OP-RU + IL-12/IL-18 (combination treatment) and 4. DMSO control (untreated condition).

**Fig 1:**
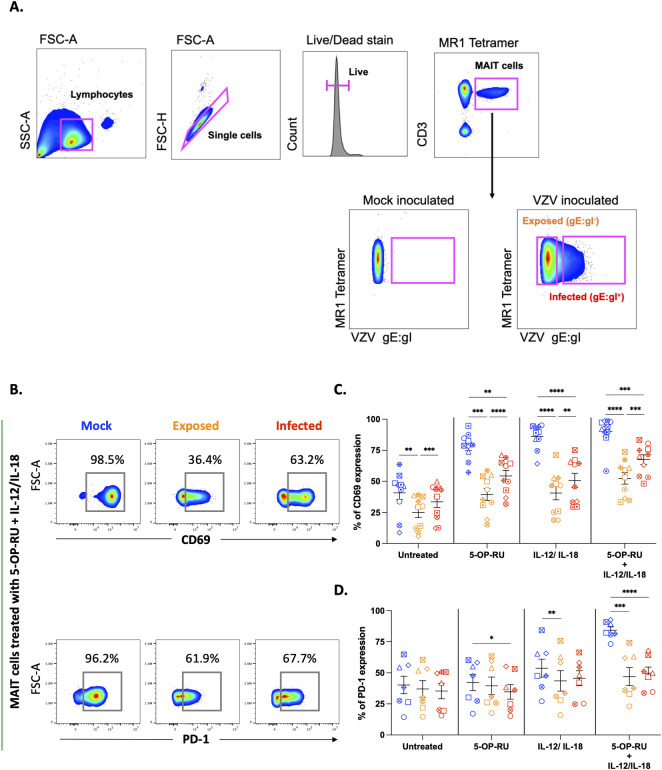
VZV impairs MAIT cell activation. Human PBMCs were inoculated with mock or clinical VZV isolate (VZV-S) infected ARPE-19 epithelial cells for one day. PBMCs were removed from co-culture, before treating with different stimulations as specified, and then analysed by flow cytometry. **(A)** Representative flow cytometry plots depict the gating strategy to identify MAIT cells in PBMCs as CD3^+^ and MR1-Tetramer^+^. Following MAIT cell identification, surface VZV glycoprotein (g)E:gI complex staining was used to subgroup MAIT cells as either Infected (gE:gI^+^) or Exposed (gE:gI^-^). **(B)** Flow cytometry plots depict surface expression of CD69 and PD-1 of mock, exposed and infected MAIT cells in response to 5-OP-RU + IL-12/IL-18 treatment. **(C and D)** Graphs show frequency of CD69 **(C)** and PD-1 **(D)** expression of mock (blue), exposed (orange) and infected (red) MAIT cells to treatments as specified with symbols representing individual donors and mean and SEM indicated by the bars. Statistical analysis comparing CD69 **(C)** and PD-1 **(D)** expression between mock, exposed and infected MAIT cells within each treatment group was performed via Two-Way repeated measures ANOVA (n = 10). *p<0.05, **p<0.01, ***p<0.001, ****p<0.0001.

MAIT cells were identified via co-staining of 5-OP-RU loaded MR1 tetramer and CD3 (Fig 1A). Consistent with previous literature, we detected MAIT cell numbers at an average of 2.2% of live T cells; with no difference in frequency observed between mock and VZV co-cultured MAIT cells (S1A Fig). A widely utilised readout of VZV infection being the detection of surface viral glycoprotein E (gE):gI complex (expressed late in VZV replication cycle) [4,8–10,38,39] was employed to classify MAIT cells as either virally infected or exposed (Fig 1A). We observed a significant increase in the percentage of gE:gI expression in TCR ligand (5-OP-RU) treated MAIT cells (mean 22.5%) compared to untreated MAIT cells (mean 9.8%) (S1B Fig), suggesting that TCR stimulation may promote a greater infection of MAIT cells.

Using surface CD69 and PD-1 expression as markers of early activation, we observed a robust expression of CD69 and PD-1 in mock co-cultured MAIT cells for TCR ligand, cytokine and combination treatment groups (Fig 1B and 1C). Comparatively, a significant reduction of both CD69 and PD-1 expression was demonstrated by VZV infected MAIT cells across all treatment groups (Fig 1C). Interestingly, CD69 expression of VZV exposed and infected MAIT cells was significantly lower compared to mock in the untreated condition (Fig 1C), suggesting that VZV may alter the underlying response potential of MAIT cells.

Strikingly, VZV exposed MAIT cells exhibited significantly lower activation levels compared to both mock and VZV infected MAIT cells across all treatment groups (Fig 1C). To examine any relationship between the magnitude of MAIT cell infection and activation status: we performed correlation analysis between the fluorescence intensity of gE:gI staining and CD69 expression and found no correlation.

Viral infection of MAIT cells, can result in apoptotic programming and death; as seen with measles virus infection [40]. Therefore, we examined MAIT cell apoptosis levels following VZV inoculation by utilising a well-established flow cytometric based detection of intracellular cleaved Caspase-3 as a readout for apoptosis [41]. We found that VZV exposed and infected MAIT cells did not exhibit significantly greater levels of apoptosis compared to mock (S2A and S2B Fig). Therefore indicating the observed impairment of MAIT cell activation by VZV to not be likely a result of apoptotic programming.

Taken together, these results suggest VZV drives an impairment of MAIT cell activation in both the directly infected (gE:gI^+^) as well as exposed (gE:gI^-^) cells. Furthermore, this impairment was consistently observed in TCR-dependent, cytokine driven and combination treatment groups, suggesting a potentially global paralysis of MAIT cell response to several distinct modalities of stimulation.

### VZV co-cultured MAIT cells are functionally refractory to both TCR-dependent and cytokine driven stimulation

**Fig 2:**
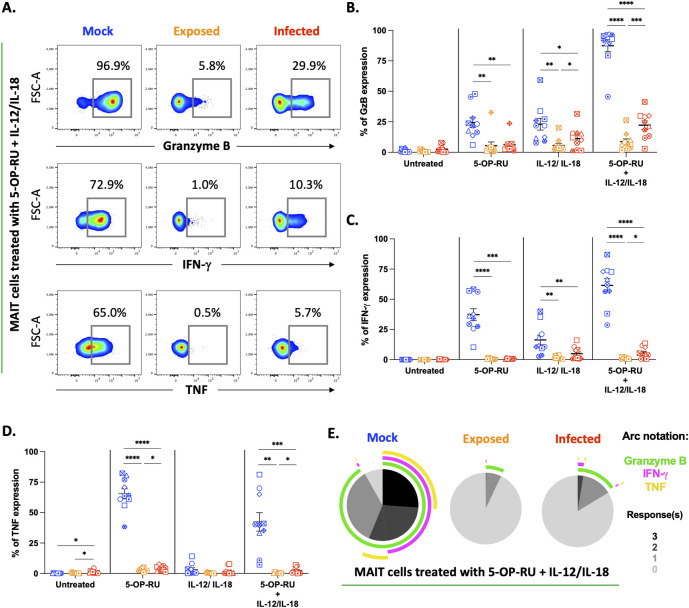
VZV co-cultured MAIT cells are functionally refractory to both TCR dependent and cytokine driven stimulation. Human PBMCs were inoculated with mock or clinical VZV isolate (VZV-S) infected ARPE-19 epithelial cells for one day. PBMCs were removed from co-culture, before treating with different stimulations as specified, and then analysed by flow cytometry. **(A)** Flow cytometry plots depict intracellular expression of granzyme B, IFN-γ and TNF of mock, exposed and infected MAIT cells in response to 5-OP-RU + IL-12/IL-18 treatment. **(B,C and D)** Graphs show frequency of granzyme B **(B)**, IFN-γ **(C)** and TNF **(D)** expression of mock (blue), exposed (orange) and infected (red) MAIT cells to treatments as specified with symbols representing individual donors and mean and SEM indicated by the bars. Statistical analysis comparing Granzyme B **(B)**, IFN-γ **(C)** and TNF **(D)** expression between mock, exposed and infected MAIT cells within each treatment group was performed via Two-Way repeated measures ANOVA (n = 10). *p<0.05, **p<0.01, ***p<0.001, ****p<0.0001. **(E)** SPICE pie charts show the proportion of responses by mock, exposed and infected MAIT cells to 5-OP-RU + IL-12/IL-18 stimulation, based on the combinations of granzyme B, IFN-γ and TNF expression. Pie slices indicate the number of responses (0–3) (key, bottom right). Arcs depict the markers detected for each response (key, top right). SPICE data represents the mean of five donors.

Next, we sought to determine whether the lack of activation in VZV co-cultured MAIT cells corresponds to a reduction of cytokine and granzyme expression. Intracellular expression of granzyme B, IFN-γ, and TNF was assessed by flow cytometry to evaluate MAIT cell pro-inflammatory functional responses. In mock, VZV exposed and VZV infected MAIT cells, minimal granzyme B, IFN-γ and TNF expression was observed in the untreated condition (Fig 2). Stimulation of mock MAIT cells with combination treatment resulted in the most notable increase of granzyme B expression (83-fold increase compared to untreated condition) (Fig 2B and 2E). Increase of granzyme B expression was also observed for VZV infected MAIT cells across all treatment conditions compared to the untreated condition (Fig 2E). However, compared to mock, VZV infected MAIT cells demonstrated a significantly reduced ability to express granzyme B across all modalities of activation (Fig 2B). Expression of IFN-γ was the highest in combination treated mock MAIT cells (61-fold increase compared to untreated condition), which was also observed albeit to a lesser extent in VZV infected MAIT cells (5-fold increase compared to untreated condition). Across all stimulation conditions, VZV infected MAIT cells exhibited a significant inhibition of IFN-γ expression (Fig 2C). Interestingly, TCR ligand treatment of mock MAIT cells induced the greatest level of TNF expression (65-fold increase compared to untreated condition), whilst cytokine treatment of MAIT cells failed to significantly induce TNF expression as previously observed [22] (Fig 2D and 2G). Again, VZV infected MAIT cells failed to significantly express TNF in any stimulation groups when compared to mock (Fig 2D). Interestingly, a minor but significant upregulation of TNF was observed in the untreated condition by VZV infected MAIT cells (mean 1.1%) compared to mock (mean 0.17%) (Fig 2D).

Strikingly, VZV exposed MAIT cells demonstrated the greatest impairment in expressing granzyme B, IFN-γ, and TNF compared to mock across TCR dependent and TCR independent forms of stimulation (Fig 2). To further characterise whether the VZV exposed MAIT cell subpopulation was *bona-fide* exposed or in early infection stages, we infected PBMCs with VZV inoculum for 24 hours before FACS sorting on gE:gI negative MAIT cells and then culturing for 24 and 48 hours in isolation (S3A Fig). We observed that almost all gE:gI negative MAIT cells remained gE:gI negative at both 24 and 48 hours suggesting these cells to be likely resistant to productive infection (S3B Fig).

Next, we wanted to investigate whether suppression of MAIT cell cytokine expression may extend to lower infectious doses. Therefore, we inoculated PBMCs with a range of viral inoculum: PBMC ratios (1:5, 1:10, and 1:20). In line with our previous study: we observed a ratio-dependent infection of MAIT cells, with the 1:20 dose generating a significantly lower level of infection compared to the 1:5 dose [38], and this was consistent across all MAIT cell stimulation conditions (S4A Fig). Furthermore, we found that even at the lowest ratio of 1:20, both exposed and infected MAIT cells demonstrated significantly lower levels of CD69 and IFN-γ co-expression compared to mock infection across all stimulation conditions (S4B and S4C Fig).

Detection of IFN-γ and TNF co-expression in mock MAIT cells was only seen in the TCR ligand (mean 31.7%) and combination (ligand plus cytokines) treatment groups (mean 37.7%) (S5A and S5B Fig). In contrast, both VZV infected and exposed MAIT cells demonstrated almost a complete absence of IFN-γ and TNF co-expression for both ligand TCR and combination treatment groups (S5B Fig).

Furthermore, through Boolean gating of functional marker co-expression we utilised SPICE (Simplified Presentation of Incredibly Complex Evaluations) analysis to quantitatively describe the lack of polyfunctional response observed in bystander and infected MAIT cells compared to mock. SPICE analysis revealed that an average of 6% of TCR ligand stimulated mock MAIT cells co-expressed granzyme B, IFN-γ, and TNF (S5C Fig), whilst 26.4% of combination stimulated mock MAIT cells expressed all three functional markers (Fig 2E). This polyfunctional response to TCR as well as combination stimulation was notably absent in both VZV exposed and infected MAIT cells (Fig 2E). Collectively, we observed a profound inability for both VZV exposed and infected MAIT cells to express several functional markers in response to TCR dependent, TCR-independent or combined stimulation.

### Differential expression of transcription factors in unstimulated and stimulated VZV co-cultured MAIT cells

**Fig 3:**
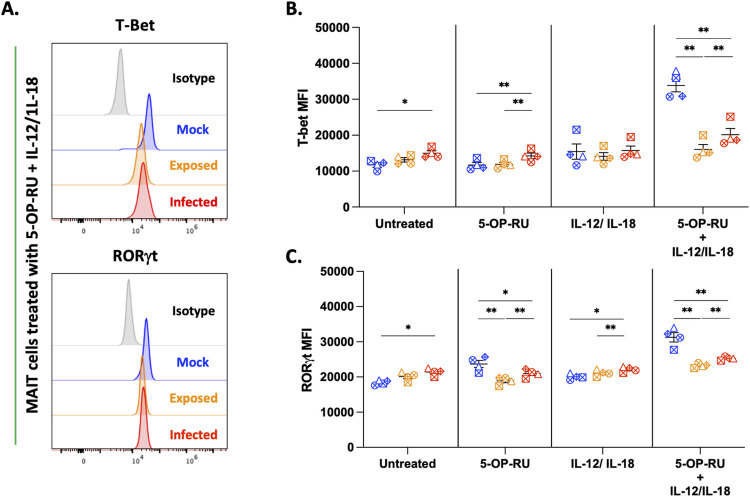
Differential expression of transcription factors in unstimulated and stimulated VZV and mock co-cultured MAIT cells. Human PBMCs were inoculated with mock or clinical VZV isolate (VZV-S) infected ARPE-19 epithelial cells for one day. PBMCs were removed from co-culture, before treating with different stimulations as specified, and then analysed by flow cytometry. **(A)** Histograms depict intra-nuclear expression of T-bet and RORγt in mock, exposed and infected MAIT cells in response to 5-OP-RU + IL-12/IL-18 treatment. **(B and C)** Graphs show mean fluorescence intensity (MFI) of T-bet **(B)** and RORγt **(C)** of mock (blue), exposed (orange) and infected (red) MAIT cells to treatments as specified with symbols representing individual donors and mean and SEM indicated by the bars. Statistical analysis comparing T-bet **(B)** and RORγt **(C)** MFI between mock, exposed and infected MAIT cells within each treatment group was performed via Two-Way repeated measures ANOVA (n = 4). *p<0.05, **p<0.01.

**Fig 4:**
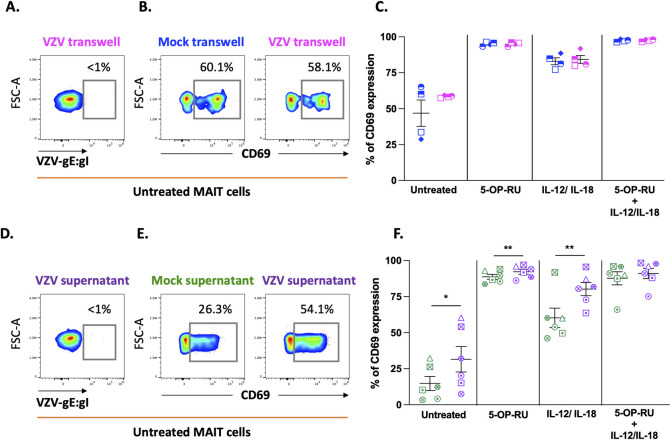
VZV impairment of MAIT cells is contact dependent and not mediated through soluble factors. **(A, B and C)** Human PBMCs were separated using a transwell system from mock or clinical VZV isolate (VZV-S) infected ARPE-19 epithelial cells and co-incubated for one day. PBMCs were removed from transwell insert, treated with different stimulations as specified, and then analysed by flow cytometry. **(A)** Flow cytometry plot depicts surface VZV-gE:gI expression of MAIT cells following transwell co-incubation. **(B)** Flow cytometry plots depict surface CD69 expression of untreated MAIT cells co-incubated with either mock or VZV infected cells with a transwell. **(C)** Graph shows frequency of CD69 expression of mock (blue) and VZV (magenta) co-incubated MAIT cells to treatments as specified with symbols representing individual donors and mean and SEM indicated by the bars. Statistical analysis comparing CD69 expression in mock and VZV co-incubated MAIT cells within each treatment group was performed via Šídák’s multiple comparisons test (n = 4). **(D,E and F)** Human PBMCs were incubated for one day with supernatants derived from mock or VZV co-culture with PBMCs. PBMCs were then treated with different stimulations as specified, and analysed by flow cytometry. **(D)** Flow cytometry plot depicts surface VZV-gE:gI expression of MAIT cells incubated with VZV-PBMC derived supernatant. **(E)** Flow cytometry plots depict surface CD69 expression of untreated MAIT cells incubated with either mock-PBMC or VZV-PBMC co-culture derived supernatant. **(F)** Graph shows frequency of CD69 expression of mock (green) and VZV (purple) supernatant incubated MAIT cells to treatments as specified with symbols representing individual donors and mean and SEM indicated by the bars. Statistical analysis comparing CD69 expression of mock or VZV supernatant incubated MAIT cells within each treatment group was performed via Šídák’s multiple comparisons test (n = 6). *p<0.05, **p<0.01.

The observation that VZV exposed and infected MAIT cells failed to *denovo* express key cytokine and cytolytic markers in response to various stimuli, raised the possibility that VZV potentially targets upstream regulators of protein synthesis such as transcription factors. Thus we assessed the expression of transcription factors T-bet and RORγt which serve as master regulators of the Th1 and Th17 response respectively. Interestingly, in the unstimulated condition we observed significantly increased mean fluorescence intensity (MFI) of T-bet and RORγt in VZV infected MAIT cells compared to mock (Fig 3A, 3B and 3C). Furthermore, an upregulation of T-bet MFI was observed in ligand TCR stimulated VZV infected MAIT cells (Fig 4B), whilst increased RORγt MFI was also observed in cytokine stimulated VZV infected MAIT cells (Fig 3C). In accordance with the activation and cytokine expression data, combination stimulation induced the greatest expression of T-bet and RORγt in mock MAIT cells (Fig 3B and 3C). Again, both VZV exposed and infected MAIT cells failed to upregulate T-bet and RORγt expression and demonstrated significantly lower MFI compared to combination stimulated mock MAIT cells (Fig 3B and 3C). Interestingly, VZV exposed MAIT cells exhibited lower T-bet and RORγt expression compared to VZV infected MAIT cells across all treatment groups (Fig 3B and 3C). This corresponds with earlier data (Figs 1 and 2) which also demonstrated consistently lower activation, cytokine and granzyme production by VZV exposed MAIT cells compared to those infected. The failure of VZV exposed and infected MAIT cells to upregulate T-bet and RORγt after combination stimulation correlates with the lack of cytokine and granzyme expression in response to stimulation. However, the lack of a complete abrogation of transcription factor expression suggests that VZV targets additional mechanisms in the T cell activation pathway to regulate MAIT cell functional response.

### VZV impairment of MAIT cells is contact dependent and not mediated by soluble factors

The remarkable extent to which VZV exposed MAIT cells were functionally unresponsive to stimulation prompted the possibility that the inhibition of MAIT cells may be mediated by soluble factors within the viral co-culture supernatant. To test this, PBMCs were separated from mock or VZV inoculum by a transwell membrane and then stimulated through TCR dependent and independent modalities as previously described. The absence of gE:gI staining in PBMCs demonstrated a lack of infection by VZV (Fig 4A). This was not surprising as VZV is highly cell-associated *in vitro* with extremely limited release of cell-free virions, therefore requiring cell-cell contact for transmission of virus [42]. Furthermore, we observed no difference in CD69 expression between mock and VZV co-cultured MAIT cells across all treatment conditions (Fig 4B and 4C), suggesting that cell-contact with viral inoculum is required for inhibition of MAIT cell response. However, this system does not exclude the possibility of an inhibitory secreted factor expressed after contact between viral inoculum and PBMCs. We therefore collected supernatant generated after 1 day of direct cell contact between mock or viral inoculum with PBMCs and incubated fresh PBMCs with mock or viral supernatant for 24 hours. As expected, incubation with viral supernatant did not result in infection of PBMCs (Fig 4D). Interestingly, viral supernatant induced greater expression of CD69 expression by MAIT cells in the untreated, ligand TCR and cytokine stimulated conditions compared to mock supernatant (Fig 4E and 4F). Taken together, the data suggest that impairment of MAIT cell response to stimulation by VZV is contact dependent and not mediated by soluble factors.

### VZV impairs cytolytic potential of MAIT cells towards bacterially treated cells

**Fig 5:**
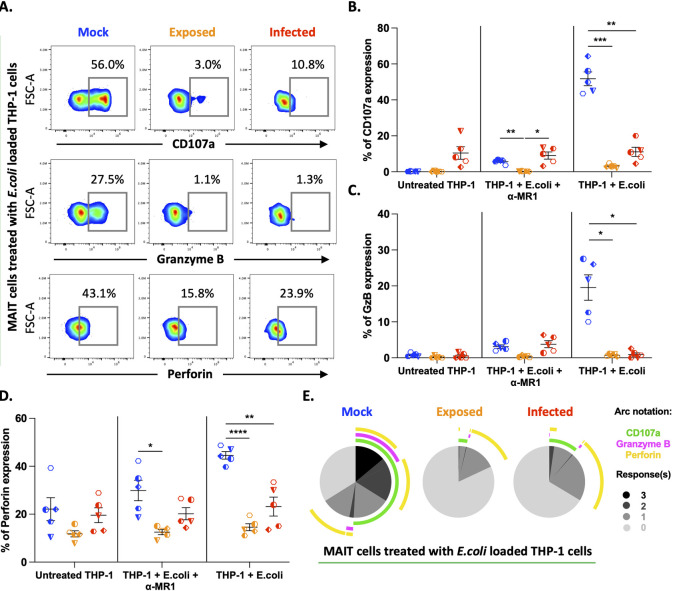
VZV impairs MAIT cell cytolytic potential towards bacterially stimulated target cells. **(A,B,C,D and E)** Human PBMCs were inoculated with mock or clinical VZV isolate (VZV-S) infected ARPE-19 epithelial cells for one day. PBMCs were removed from co-culture, before treating with different stimulations as specified, and then analysed by flow cytometry. **(A)** Flow cytometry plots depict surface expression of CD107a and intracellular expression of granzyme B and perforin by mock, exposed and infected MAIT cells in response to treatment with *E*. *coli* loaded THP-1 cells. **(B,C and D)** Graphs show frequency of CD107a **(B)**, Granzyme B **(C)** and Perforin **(D)** expression of mock (blue), exposed (orange) and infected (red) MAIT cells to treatments as specified with symbols representing individual donors and mean and SEM indicated by the bars. Statistical analysis comparing CD107a **(B)**, Granzyme B **(C)** and Perforin **(D)** expression between mock, exposed and infected MAIT cells within each treatment group was performed via Two-Way repeated measures ANOVA (n = 6). *p<0.05, **p<0.01, ***p<0.001, ****p<0.0001. **(E)** SPICE pie charts show the proportion of responses by mock, exposed and infected MAIT cells to *E*. *coli* loaded THP-1 cells based on the combinations of CD107a, Granzyme B, and perforin expression. Pie slices indicate the number of responses (0–3) (key, bottom right). Arcs depict the markers detected for each response (key, top right). SPICE data represents the mean of five donors.

Next, we wanted to determine whether VZV impairment of MAIT cell functions extended to a more physiologically reflective model of MAIT cell response to intact bacteria. We examined the expression of CD107a, granzyme B and perforin to evaluate the cytolytic potential of VZV co-cultured MAIT cells in response to intact bacteria. Briefly, THP-1 cells (antigen presenting cell line) were loaded with partially fixed *E*. *coli* for two hours, with the addition of either MR1 blocking antibody or respective isotype control antibody in the final hour of loading to control for TCR driven activation of MAIT cells. Mock or VZV inoculated PBMCs were then co-cultured with loaded THP-1 cells for 6 hours. Compared to both untreated and MR1 blocking condition, *E*. *coli* treated mock MAIT cells demonstrated a 51-fold increase in CD107a expression (Fig 5A and 5B). Contrastingly, VZV infected MAIT cells maintained consistent expression of CD107a across all treatment conditions, with no observed upregulation with *E*. *coli* treatment (Fig 5B). Again, *E*. *coli* treatment induced the greatest expression of Granzyme B in mock MAIT cells (19.6-fold increase compared to untreated), whilst this upregulation was completely absent in VZV infected MAIT cells (Fig 5C). Without stimulation, mock MAIT cells expressed perforin (mean 22.1%, untreated condition), which increased in both the MR1 blocking condition (mean 29.9%) and *E*. *coli* treatment (mean 44.5%) (Fig 5D). Conversely, VZV infected cells also expressed perforin in unstimulated cells (mean 19.6%, untreated condition) however, this did not increase with *E*. *coli* treatment (mean 23.6%) or MR1 blockade (mean 20.2%) (Fig 5D). VZV exposed MAIT cells exhibited the greatest inhibition of CD107a and Granzyme B expression in all treatment conditions (Fig 5B and 5C). Interestingly, VZV exposed MAIT cells expressed lower perforin at when unstimulated (mean 11.8%, untreated condition), and demonstrated a failure to upregulate across stimulation conditions (Fig 5D). Strikingly, SPICE analysis revealed that perforin expression in *E*. *coli* treated, VZV exposed and infected MAIT cells did not correspond to CD107a expression, therefore suggesting a lack of degranulation and release of perforin (Fig 5E). Overall, we observed defective cytolytic potential of VZV exposed and infected MAIT cells to intact bacterial presence.

## Discussion

This study identifies a multifaceted immune evasion strategy utilised by VZV to restrict MAIT cell responses to TCR dependent and TCR-independent stimuli. Specifically, VZV was found to compromise activation, cytokine expression and cytolytic capacity of human MAIT cells.

Successful infection of the host involves effectively managing several complex trans-kingdom interactions between the pathogen, host and resident microbiome. Varicella manifests as cutaneous vesicular lesions, which disrupt the normal skin architecture at these sites within the host. Importantly, this potentially permits translocation of MAIT cell-activating resident riboflavin-synthesising microbes. Indeed, the most common complication from severe VZV infection is secondary bacterial infection from species such as *Staphylococcus aureus* (*S*.*aureus*) [13,43]. Within this co-localised microenvironment of active riboflavin biosynthesis and viral replication, MAIT cells can rapidly produce pro-inflammatory cytokines, including IFN-γ and TNF, which poses a potential threat to VZV pathogenesis. This is exemplified by several reports that demonstrate that IFN-γ and TNF mediated control of VZV replication and spread [44]. Indeed, recent reports also describe increased reactivation of VZV in patients undergoing anti-TNF treatment [45]. Remarkably, we found that both VZV exposed and infected populations targetted several effector arms of MAIT cells such as pro-inflammatory cytokine expression and cytolytic potential in response to both TCR dependent and cytokine dependent stimulation. Therefore, revealing a profound ability of VZV to suppress MAIT cell polyfunctional responses towards several distinct modalities of stimulation.

Furthermore, we demonstrated this impairment of MAIT cell responses to not only the purified MAIT TCR ligand, but also in response to intact riboflavin- synthesising bacteria. Given our previous report characterising VZV modulation of MR1 antigen presentation [37], it is possible that the restriction of TCR driven MAIT cell functionality could be indirectly attributed to an impaired ability of surrounding APCs in the PBMC-inoculum co-culture system to present 5-OP-RU. However, the functional inhibition of MAIT cells in response to exogenously added THP-1 cells pre-loaded with *E*.*coli* strongly suggests VZV dysregulation of TCR driven MAIT cell response to be a direct consequence of exposure and infection.

It remains to be shown whether the flow cytometry based assessment of functional cytolytic markers within this study translates to distinct functional outcomes such as impaired direct lysis of bacterially challenged APCs by exposed and infected MAIT cells. It is important to note however that without extensive antigen priming, prolonged expansion and substantial prior activation, *ex-vivo* MAIT cells are poorly cytolytic and display limited target cell killing [23,46–51]. Therefore, the extensive demands required for cytolytically arming and licensing MAIT cell killing may prove challenging due to the timescale of the infection model utilised within this current study. Thus, further studies are required to functionally illuminate the potential impairment of MAIT cell killing capacity presented within this study.

Previous landmark studies have demonstrated redirection of phosphorylation cascades in key TCR signalling proteins by VZV [8]. In particular, VZV infected tonsillar T cells exhibit increased Zap70 phosphorylation [8]; which is a critical signalling event for T cell activation following TCR engagement [52]. Interestingly, increased Zap70 phosphorylation by VZV did not result in typical downstream phosphorylation of proteins leading to cytokine production such ERK1/2, but instead non-classical phosphorylation cascades associated with cell proliferation pathways [8]. Given the conservation of TCR signalling cascade across T cells, it is likely VZV employs a similar restructuring of MAIT TCR signalling to prevent a functional response. Additionally, the closely related virus: herpes simplex virus type 1 (HSV-1) encodes protein kinase Us3 which partially inhibits TCR signalling through disrupting activation of key pathway protein LAT [53]. However, the VZV encoded homolog ORF66 is critical for T lymphotropism [7]. Therefore, investigating the impact of ORF66 on MAIT TCR signalling presents several complexities.

Whilst no reports to date have identified a viral encoded MAIT TCR ligand, MAIT cells play a role in several viral infections by responding to cytokine production from infected host cells [34–36]. Similarly, we observed increased MAIT cell activation in response to incubation with VZV-PBMC supernatant, therefore strongly suggesting an underlying impact of VZV to also target cytokine driven activation of MAIT cells. We found that VZV infected MAIT cells remained unresponsive to IL-12/IL-18 stimulation with abrogated activation, cytokine and granzyme B production. It will be important to investigate whether VZV directly targets the expression of IL-12 and IL-18 receptor expression on MAIT cells to mediate this impairment of cytokine driven response. Previous work has demonstrated that VZV prevents the nuclear translocation of nuclear factor κB (NF-κB) in immature dendritic cells [54]. Given that both cytokine and TCR mediated stimulation of T cells converges through the NF-κB pathway [55], it is possible that VZV disruption of NF-κB translocation in MAIT cells may explain the global restriction of MAIT cell functional response towards TCR, cytokine and combination stimulation.

It is likely that a predetermined pool of VZV susceptible MAIT cells exists given that VZV antigen negative cells did not become VZV antigen positive 24 and 48 hours after sorting. This correlates with our previous finding that VZV infection of MAIT cells does not significantly increase from 24-72hpi [38]. Both findings support the notion that a predominant majority of MAIT cells do not support productive infection by VZV. This pattern of infection susceptibility has also previously been observed in studies on VZV permissiveness of NK cells and T cells [8,9]. Strikingly, despite the inability to successfully infect the majority of MAIT cells in our co-culture system, we found that VZV exposed MAIT cells consistently exhibited the greatest impairment of activation, transcription factor and functional marker expression in response to TCR dependent and TCR independent forms of stimulation. Lack of productive infection however does not preclude the possibility of direct viral contact and entry of VZV into MAIT cells. Previous studies have shown that contact between VZV inoculum and plasmacytoid dendritic cells (pDCs) impaired their ability to express IFN-α [6], whilst inhibition of NK functionality by VZV is also contact dependent [10]. These findings are congruent with HSV-1 dysregulation of TCR signalling in both immortalised T cells and invariant natural killer T (iNKT) cells, which was revealed to be dependent on viral entry not infection [56,57]. Future studies utilising anti-viral drugs such as acyclovir [58], which permit viral entry but restrict *de novo* viral gene expression, will shed light on whether suppression of MAIT cell response in VZV exposed cells is mediated by viral entry.

There are now several reports that characterise decreased functional capacities of MAIT cells as an outcome of various infections. Increased conjugated bilirubin levels released from liver damage during chronic HBV infection drives exhaustion and impairs TCR driven MAIT cell activation and proliferation [59]. Altered MAIT cell phenotypes observed during other liver damaging disease such as HCV infection [60] suggests that increases in conjugated bilirubin levels may drive this MAIT cell disruption as seen in conventional CD4^+^ T cells [61]. Studies have now also reported a persistent decline and exhaustion of MAIT cells during HIV infection [62,63]. HIV infection drives sustained IFN-α expression consequently inducing a counter-balancing IL-10 driven immune-suppressive response by monocytes [64]. Critically, this increased IL-10 production impairs TCR driven CD107a and granzyme expression by MAIT cells in response to *E*. *coli* stimulation [64]. Furthermore, a recent study demonstrated activation induced pyroptosis of MAIT cells in response to HIV virions [63], which may potentially explain the consistent observation of declining circulating MAIT cells in HIV patients.

Overall, there is a growing appreciation that MAIT cells play an important role in the control of bacterial and viral infections. Our study characterises a direct immunoevasive strategy employed by a pathogen to counteract MAIT cell responses. Specifically, we demonstrate that exposure to, and infection by, VZV causes a profound suppression of MAIT cell activation and functional response, both to TCR-dependent and -independent forms of stimulation. Future mechanistic insights into the restriction of MAIT cell responses by VZV could potentially be harnessed for bio-active therapeutic alternatives in treating chronic inflammatory settings such as rheumatoid arthritis, multiple sclerosis, and inflammatory bowel diseases where MAIT cells play an immunopathological role [65]. The conserved nature of MAIT cell activation and downstream functional response drives a selective evolutionary pressure for a diverse range of human pathogens. Therefore, it is likely that the restriction of MAIT cell response is not a strategy unique to VZV, but one that is potentially also employed by other pathogens to varying degrees. Thus, our findings predicate an exploration into how pathogens can directly control and compromise MAIT cell mediated host responses.

## Materials and methods

### Ethics statement

All blood work was performed in the accordance with The University of Sydney Human Research Ethics Committee approval. All blood donations were obtained under agreement with the Australian Red Cross Lifeblood service and all donors provided written informed consent.

### Human blood

Healthy peripheral blood mononuclear cells (PBMCs) were isolated from buffy coats using density gradient centrifugation with Ficoll-Paque PLUS (GE Healthcare). All buffy coats were de-identified, so any comparative data on sex and age is not available. For long term storage, PBMCs were preserved with liquid nitrogen in freezing media (90% Fetal Calf serum (FCS)) (Sigma-Aldrich) with 10% DMSO.

### Cell lines

ARPE-19 epithelial cells (ATCC) were maintained in complete DMEM (Lonza) supplemented with 10% FCS and 1% Penicillin Streptomycin (P/S) (ThermoFisher). THP-1 (human monocyte cell line, ATCC) were maintained in complete RPMI 1640 (ThermoFisher Scientific), supplemented with 10% FCS and 1% P/S. All cell lines were cultured at 37°C, 5% CO_2_.

### Short term culture of PBMCs and isolation of MAIT cells

PBMCs were thawed and washed in FCS supplemented RPMI before being maintained RPMI supplemented with 10% human serum (Sigma-Aldrich) and 1% P/S. MAIT cells were identified through fluorescent co-staining of CD3 and MR1-Tetramer positive and FACS isolated from whole PBMCs. MAIT cells were sorted to a >95% purity using BD FACSMelody (BD Biosciences) and then maintained in RPMI supplemented with 10% human serum (H/S) and 1% P/S. Where specified, VZV gE:gI antigen negative MAIT cells were also isolated to a >90% purity using BD FACSMelody (BD Biosciences) and then maintained in RPMI supplemented with 10% human serum (H/S) and 1% P/S for 24–48 hours.

### VZV inoculation of PBMCs

PMBCs were either mock or VZV inoculated through co-culture with either uninfected (mock inoculum) or VZV infected ARPE-19 cells (VZV inoculum), respectively. VZV inoculum was trypsinised after demonstrating >75% cytopathic effect and added to PBMCs at a ratio of 1:5 (ARPE-19:PBMC). Infections were performed in 12 well plates, with 2x10^6^ PBMCs in 2ml of complete RPMI medium per well. Following the addition of either mock or VZV inoculum to PBMCs, cells were spinoculated for 15 minutes at 150 x *g*, 37°C. Plates were then incubated at 37°C, 5% CO_2_ for 24 hours.

For transwell experiments, mock and VZV inoculum was seeded into the bottom chamber of a 6 well plate and separated from the top chamber containing PBMCs by a 0.4μM pore polycarbonate transwell membrane (Corning). Plates were then incubated at 37°C, 5% CO_2_ for 24 hours.

### Treatment of PBMCs with mock and viral co-culture supernatants

Following 24 hours of mock and VZV inoculation of PBMCs, supernatant was collected, spun at 460 x *g* to remove cells, and then frozen at -80°C. When required, supernatant was thawed, and diluted 1:1 with fresh complete RPMI (+10% H/S and 1% P/S) before addition to PBMCs for 24 hours.

### Preparation of bacterial stimulation

*Escherichia coli (E*. *coli)* DH5α was grown overnight in Luria-Bertani (LB) broth, washed with PBS and then partially fixed in 1% paraformaldehyde (PFA) for 3 minutes with vortexing in first 60 seconds and last 30 seconds of the fixation. After extensive washing, *E*. *coli* was added to THP-1 cells at 30 bacteria per cell (BpC) for whole PBMC stimulation assays.

### 5-OP-RU

The MAIT cell antigen 5-OP-RU was synthesised as a solution in DMSO, and its concentration quantified by NMR and MS spectra, as previously described [66]. While it is chemically stable in DMSO, and when bound to MR1 in solution, care should be taken to restrict exposure times to water during handling and dilution since 5-OP-RU rapidly transforms in aqueous media (t_1/2_ 1h, 37°C) to much less active lumazines [66]. Thus, PBMCs were treated with 5-OP-RU immediately after defrosting.

### *In vitro* MAIT cell stimulations

24 hours post-mock or -VZV inoculation, PBMCs were removed from the inoculum monolayer by gently washing off with PBS and seeded in U-bottom 96 well plates at a concentration of 1x10^6^ PBMCs/200 ml. The following treatment condition concentrations and timepoints were chosen in line with previous literature [22]. For MAIT TCR specific triggering, PBMCs were treated with 5-OP-RU (10 nM) for 6 hours. For cytokine dependent stimulation, PBMCs were treated with 50 ng/mL of IL-12 (Miltenyi) and 50 ng/mL of IL-18 (R&D Systems) for 24 h. In the combination stimulation condition, PBMCs were treated with 5-OP-RU (10 nM) and IL-12+IL-18 (each at 50 ng/mL) for 24 h. PBMCs were treated with DMSO in the untreated condition for 24 h. For stimulation with whole bacteria, THP-1 cells were pulsed with partially fixed *E*.*coli* (30 BpC) for 2 h, with the addition of either MR1 blocking antibody (5 μg) or respective isotype control (5 μg) in the final hour of pulsing. Loaded THP-1 cells were then added to PBMCs at a ratio of 1:5 (THP-1:PBMC). For assessment of cytokine content, Brefeldin A (BFA) (Biolegend) was added at 5 μg/mL for the final 4 h of the stimulation. For observation of degranulation, BFA (5 μg/mL) and Monensin (5 μg/mL) (Biolegend) was added together for the final 4 h of stimulation.

### Flow cytometry

Cells were viability stained with Live/Dead Aqua (Invitrogen), and then stained on ice for 45 minutes for the following markers: CD3—BUV395 (clone UCHT-1, BD Biosciences), CD69—PerCP Cy5.5 (clone FN50, Biolegend), PD-1—PE/Dazzle (clone EH12.2H7, Biolegend), CD161- PE/Dazzle 594 (clone HP-3G10, Biolegend), Vα 7.2—PE/Cy7 (clone 3C10, Biolegend), VZV gE:gI—Dy488 (clone SG1-1, Meridian Life Sciences), MR1-Tetramer—PE (kindly provided by A/Prof. Alexandra Corbett, University of Melbourne). Cells were then fixed in 4.2% Cytofix/Cytoperm (BD Biosciences) at room temperature for 30 minutes before staining with intracellular markers: granzyme B—PE/Cy7 (clone QA16A02), IFN-γ - BV421 (clone B27), TNF—BV785 (clone Mab11), perforin—APC/Cy7 (clone DG9); all Biologend, Caspase-3—PE (CC3) (clone C92-605, BD Biosciences). For assessment of transcription factors, cells were permeabilized with Foxp3/transcription buffer set (ThermoFisher Scientific) and then stained for: T-bet—BV421 (clone 4B10, Biolegend) and RORγt–APC (clone AFKJS-9, ThermoFisher Scientific). For degranulation staining, fluorescently conjugated anti-CD107a-APC (clone H4A3, Biolegend) or respective isotype control (Biolegend) was added to culture for the duration of stimulation. All samples were acquired on the Cytek Aurora 5 laser cytometer (Cytek), and then analysed using FlowJo v10 (TreeStar). Following exclusion of dead cells (as per viability staining) and morphological gating of lymphocytes (as per characteristic forward and side scatter), MAIT cells were identified as CD3 and MR1-Tetramer positive (Fig 1A).

### Quantification and statistical analysis

All graphs and statistical analyses were performed using GraphPad Prism V10.02. Statistical significance of marker expression between mock, exposed and infected MAIT cells was assessed using two-way repeated-measures ANOVA or paired two tailed *t* tests. Mean ± standard error of mean (SEM) is shown throughout.

## Supporting information

S1 Fig MAIT cell frequency and rate of infection across treatment conditions. Human PBMCs were inoculated with mock or clinical VZV isolate (VZV-S) infected ARPE-19 epithelial cells for one day. PBMCs were removed from co-culture, before treating with different stimulations as specified, and then analysed by flow cytometry. **(A)** Graph shows frequency of MAIT cells of live T cells after mock (blue) and VZV (red) inoculation, with symbols representing individual donors and mean and SEM indicated by the bars. Statistical analysis comparing MAIT cell frequency between mock and VZV inoculation was performed via paired *t* test (n = 19). **(B)** Graph shows frequency of gE:gI expression by MAIT cells across treatment conditions as specified, with symbols representing individual donors and mean and SEM indicated by the bars. Statistical analysis comparing gE:gI expression by MAIT cells between treatment conditions was performed via repeated measures one-way ANOVA (n = 10). *p<0.05, **p<0.01, ***p<0.001.
(TIF)

S2 Fig VZV exposed or infected MAIT cells do not exhibit significantly greater levels of apoptosis. Human PBMCs were inoculated with mock or clinical VZV isolate (VZV-S) infected ARPE-19 epithelial cells for one day. PBMCs were removed from co-culture, washed and then viability stained with Live/Dead dye. Following Live/Dead staining, PBMCs were stained for surface markers and then permeabilised with 4.2% Cytofix/Cytoperm (BD Biosciences) at room temperature for 30 minutes. Following permeabilization, PBMCs were intracellular stained for cleaved Caspase-3 (CC-3) at room temperature for 1 hour. Intracellular CC-3 expression was assessed by flow cytometry. **(A)** Flow cytometry plots depicts co-staining of Live/Dead dye with intracellular expression of CC-3 in mock, exposed and infected MAIT cells. **(B)** Graph shows frequency of CC-3 expression of mock (blue), exposed (orange) and infected (red) MAIT cells, with symbols representing individual donors and mean and SEM indicated by the bars. Statistical analysis comparing CC-3 expression between mock, exposed and infected MAIT cells was performed via Two-Way repeated measures ANOVA (n = 5). *p<0.05, **p<0.01.
(TIF)

S3 Fig Most gE:gI negative MAIT cells remain gE:gI negative. Human PBMCs were inoculated with mock or clinical VZV isolate (VZV-S) infected ARPE-19 epithelial cells for one day. PBMCs were removed from co-culture, FACS sorted for gE:gI negative MAIT cells and then cultured for 24 and 48 hours. **(A)** Representative flow cytometry plots depict the gating strategy to sort VZV exposed MAIT cells in PBMCs as CD3^+^/ MR1-Tetramer^+^/ gE:gI^-^. (B) Graph shows frequency of gE:gI expression by gE:gI negative isolated MAIT cells 24 and 48 hours post sorting. Symbols representing individual donors and mean and SEM indicated by the bars (n = 3).
(TIF)

S4 Fig VZV impairs MAIT cell response across several infectious dose ratios. Human PBMCs were inoculated with mock or clinical VZV isolate (VZV-S) infected ARPE-19 epithelial cells at varying inoculum: PBMC ratios as specified for 24 hours. PBMCs were removed from co-culture, before treating with different stimulations as specified, and then analysed by flow cytometry. **(A)** Graph shows frequency of gE:gI expression by MAIT cells across various inoculum: PBMC ratios for different treatment conditions as specified, with symbols representing individual donors and mean and SEM indicated by the bars. Statistical analysis comparing gE:gI expression by MAIT cells between different inoculum: PBMC ratios within each treatment condition was was performed via repeated measures one-way ANOVA (n = 3). *p<0.05, **p<0.01. **(B)** Flow cytometry plots depict co-expression of surface CD69 and intracellular IFN-γ for mock, exposed and infected MAIT cells in response to 5-OP-RU + IL-12/IL-18 treatment at the 1:20 inoculum: PBMC ratio. **(C)** Graphs show frequency of CD69 and IFN-γ co**-**expression of mock (blue), exposed (orange) and infected (red) MAIT cells to different treatment conditions for the various inoculum: PBMC ratios, with symbols representing individual donors and mean and SEM indicated by the bars. Statistical analysis comparing CD69 and IFN-γ co**-**expression expression of exposed and infected MAIT cells at different inoculum: PBMC ratios to mock control within each treatment group was performed via Two-Way repeated measures ANOVA (n = 3). *p<0.05, **p<0.01.
(TIF)

S5 Fig VZV impairs MAIT cell polyfunctional response. Human PBMCs were inoculated with mock or clinical VZV isolate (VZV-S) infected ARPE-19 epithelial cells for one day. PBMCs were removed from co-culture, before treating with different stimulations as specified, and then analysed by flow cytometry. **(A)** Flow cytometry plots depict intracellular co-expression of IFN-γ and TNF of mock, exposed and infected MAIT cells in response to 5-OP-RU and 5-OP-RU + IL-12/IL-18. **(B)** Graph show frequency of IFN-γ and TNF co**-**expression of mock (blue), exposed (orange) and infected (red) MAIT cells to treatments as specified with symbols representing individual donors and mean and SEM indicated by the bars. Statistical analysis comparing IFN-γ and TNF co-expression between mock, exposed and infected MAIT cells within each treatment group was performed via Two-Way repeated measures ANOVA (n = 7). *p<0.05, **p<0.01, ***p<0.001. **(C)** SPICE pie charts show the proportion of responses by mock, exposed and infected MAIT cells to 5-OP-RU stimulation, based on the combinations of granzyme B, IFN-γ and TNF expression. Pie slices indicate the number of responses (0–3) (key, bottom right). Arcs depict the markers detected for each response (key, top right). SPICE data represents the mean of seven donors.
(TIF)

S1 Data Minimal data set. (XLSX)
