# Supplementary figures and images for: Varicella Zoster Virus disrupts MAIT cell polyfunctional effector responses
Source: PLoS Pathog. 2024 Aug 7;20(8):e1012372. doi: 10.1371/journal.ppat.1012372 (PMC11305569; doi:10.1371/journal.ppat.1012372)

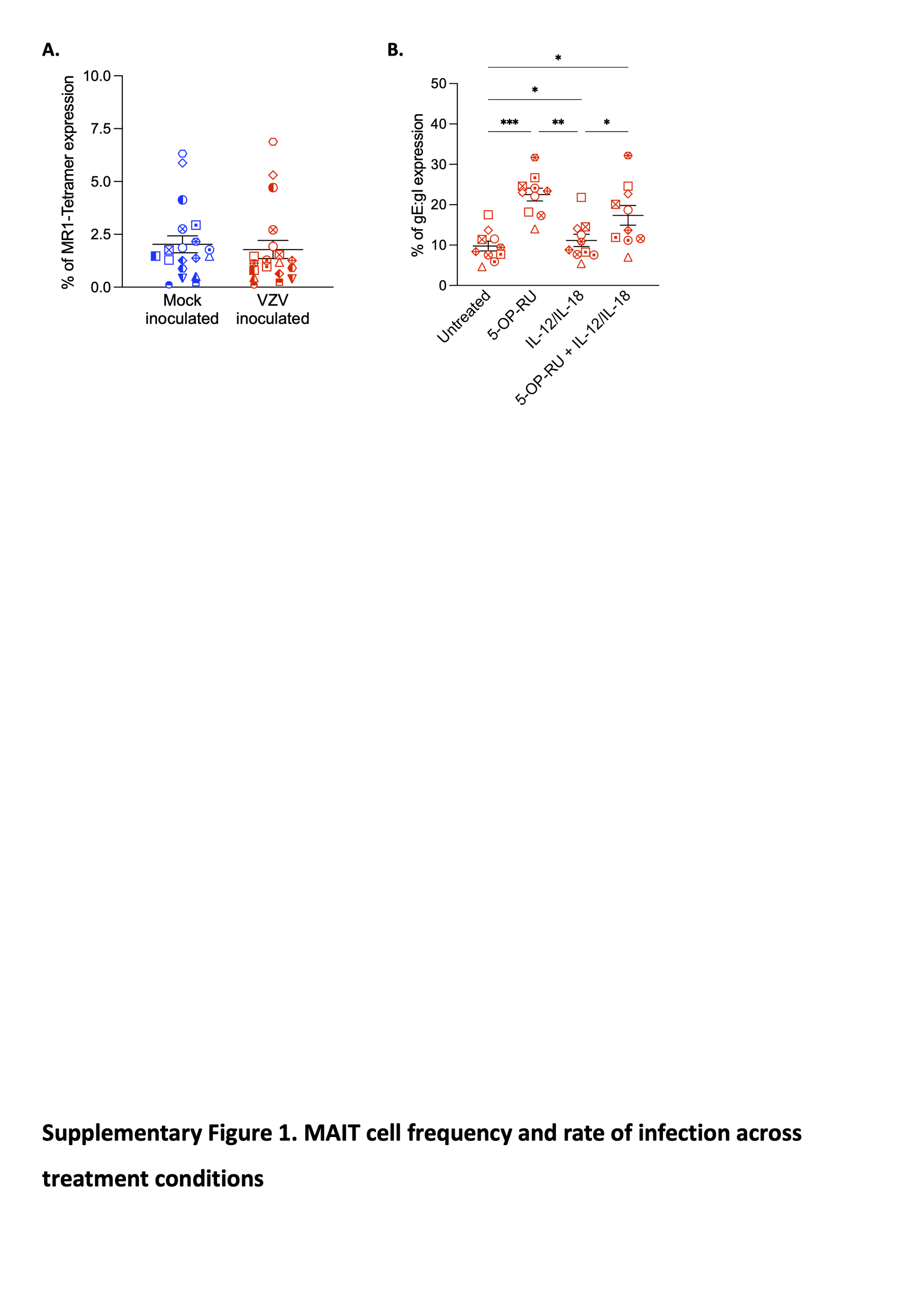

Supplement: S1 Fig — Human PBMCs were inoculated with mock or clinical VZV isolate (VZV-S) infected ARPE-19 epithelial cells for one day. PBMCs were removed from co-culture, before treating with different stimulations as specified, and then analysed by flow cytometry. (A) Graph shows frequency of MAIT cells of live T cells after mock (blue) and VZV (red) inoculation, with symbols representing individual donors and mean and SEM indicated by the bars. Statistical analysis comparing MAIT cell frequency between mock and VZV inoculation was performed via paired t test (n = 19). (B) Graph shows frequency of gE:gI expression by MAIT cells across treatment conditions as specified, with symbols representing individual donors and mean and SEM indicated by the bars. Statistical analysis comparing gE:gI expression by MAIT cells between treatment conditions was performed via repeated measures one-way ANOVA (n = 10). *p<0.05, **p<0.01, ***p<0.001. (TIF) [file ppat.1012372.s001.tif]

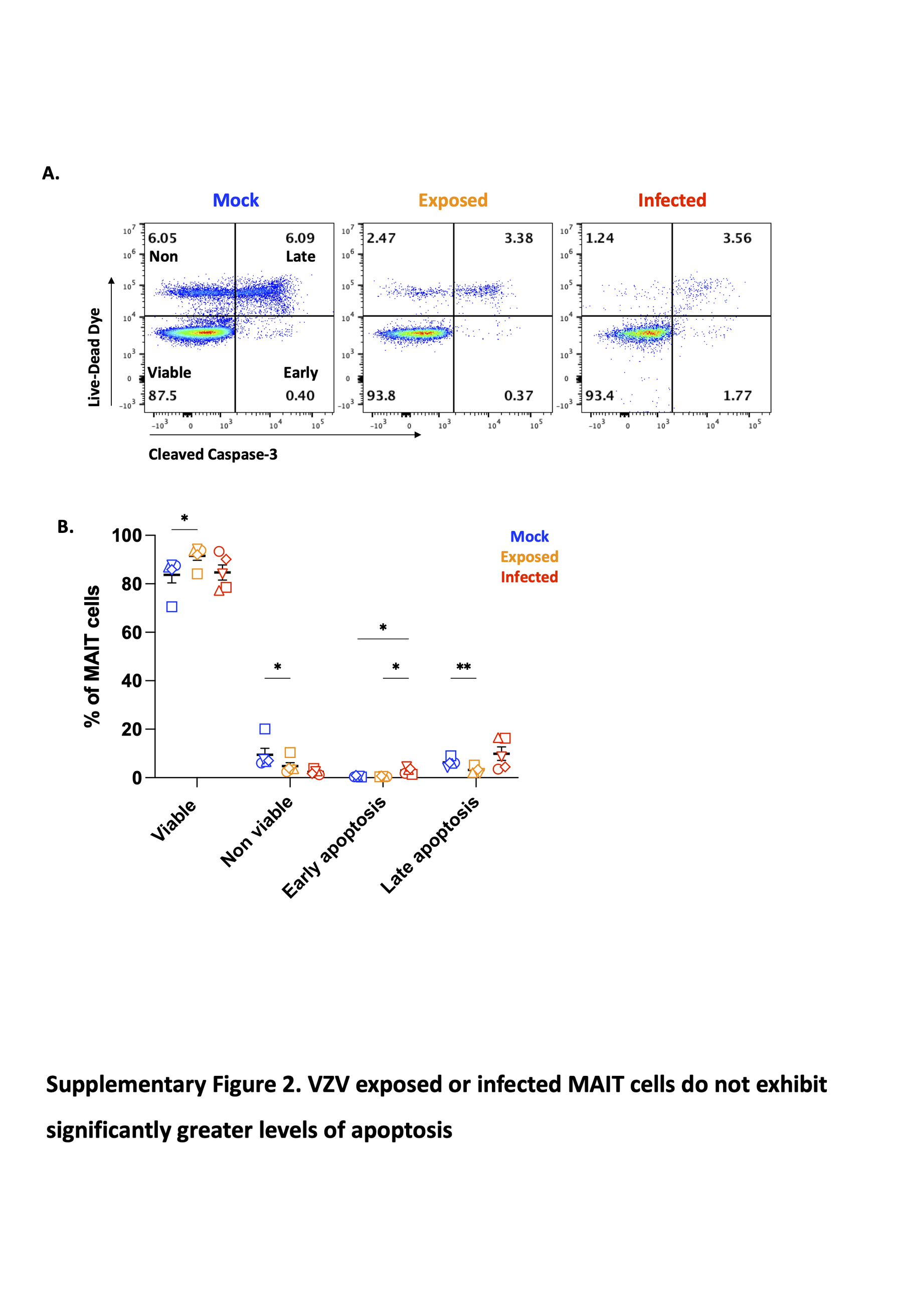

Supplement: S2 Fig — Human PBMCs were inoculated with mock or clinical VZV isolate (VZV-S) infected ARPE-19 epithelial cells for one day. PBMCs were removed from co-culture, washed and then viability stained with Live/Dead dye. Following Live/Dead staining, PBMCs were stained for surface markers and then permeabilised with 4.2% Cytofix/Cytoperm (BD Biosciences) at room temperature for 30 minutes. Following permeabilization, PBMCs were intracellular stained for cleaved Caspase-3 (CC-3) at room temperature for 1 hour. Intracellular CC-3 expression was assessed by flow cytometry. (A) Flow cytometry plots depicts co-staining of Live/Dead dye with intracellular expression of CC-3 in mock, exposed and infected MAIT cells. (B) Graph shows frequency of CC-3 expression of mock (blue), exposed (orange) and infected (red) MAIT cells, with symbols representing individual donors and mean and SEM indicated by the bars. Statistical analysis comparing CC-3 expression between mock, exposed and infected MAIT cells was performed via Two-Way repeated measures ANOVA (n = 5). *p<0.05, **p<0.01. (TIF) [file ppat.1012372.s002.tif]

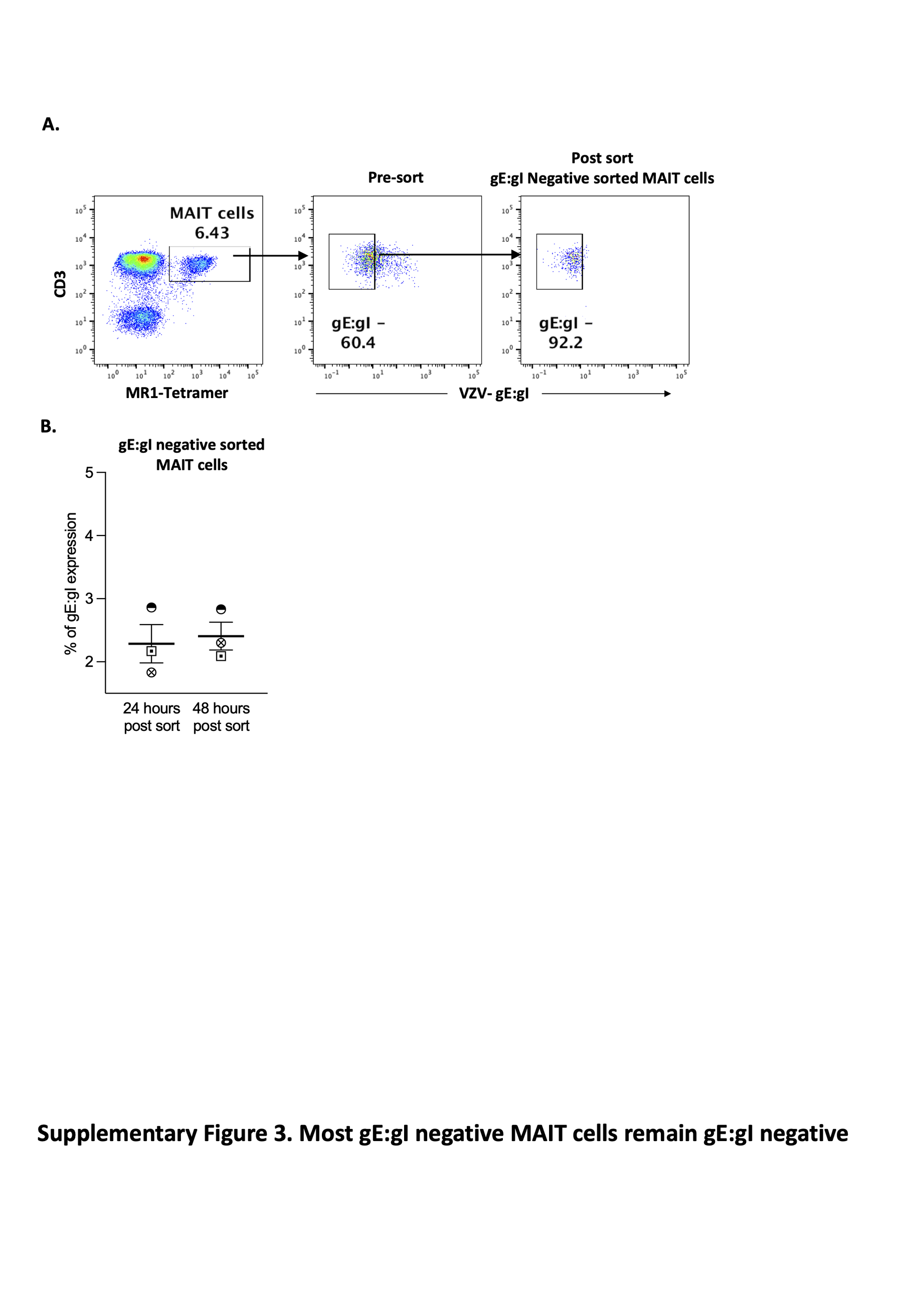

Supplement: S3 Fig — Human PBMCs were inoculated with mock or clinical VZV isolate (VZV-S) infected ARPE-19 epithelial cells for one day. PBMCs were removed from co-culture, FACS sorted for gE:gI negative MAIT cells and then cultured for 24 and 48 hours. (A) Representative flow cytometry plots depict the gating strategy to sort VZV exposed MAIT cells in PBMCs as CD3+/ MR1-Tetramer+/ gE:gI-. (B) Graph shows frequency of gE:gI expression by gE:gI negative isolated MAIT cells 24 and 48 hours post sorting. Symbols representing individual donors and mean and SEM indicated by the bars (n = 3). (TIF) [file ppat.1012372.s003.tif]

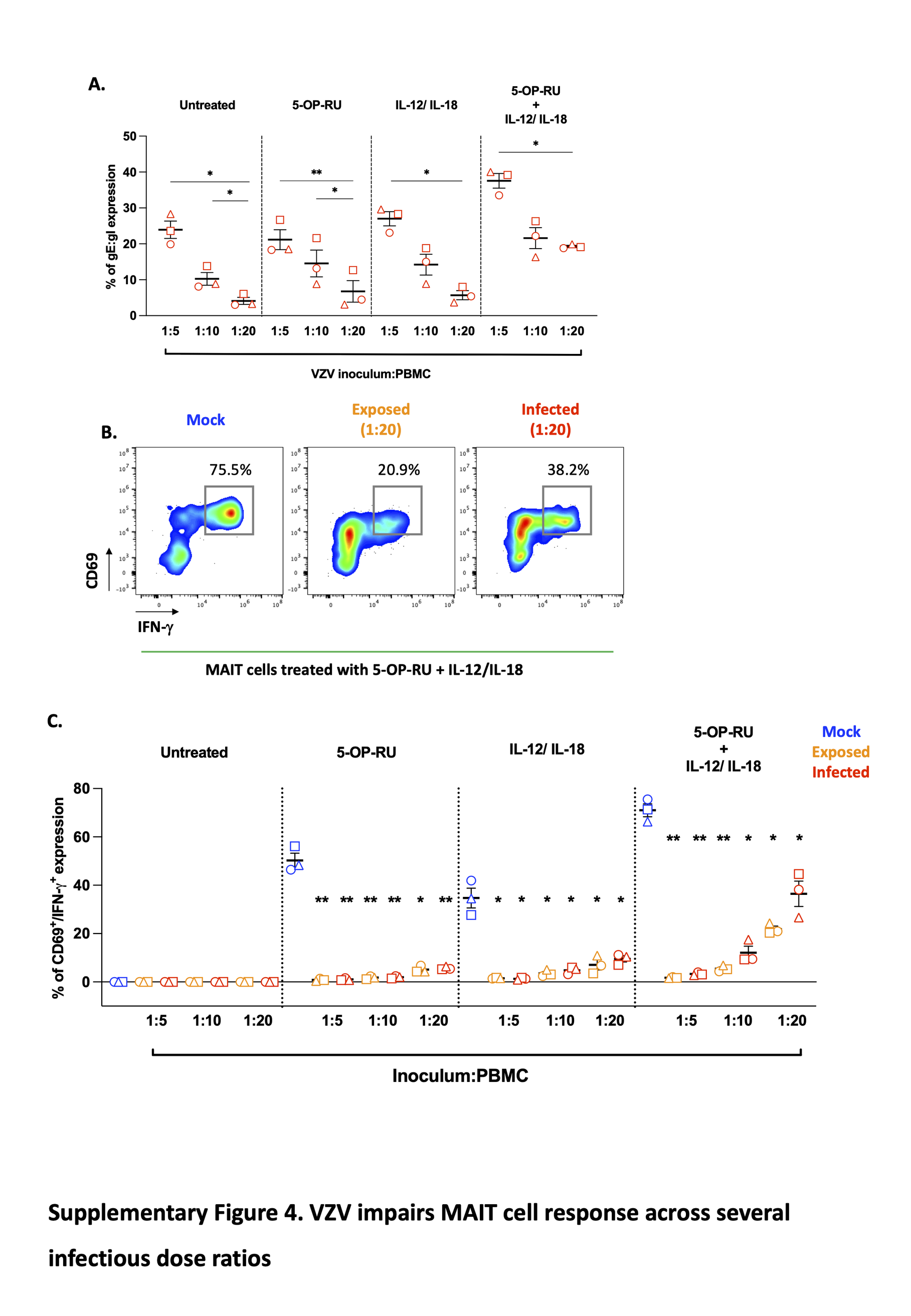

Supplement: S4 Fig — Human PBMCs were inoculated with mock or clinical VZV isolate (VZV-S) infected ARPE-19 epithelial cells at varying inoculum: PBMC ratios as specified for 24 hours. PBMCs were removed from co-culture, before treating with different stimulations as specified, and then analysed by flow cytometry. (A) Graph shows frequency of gE:gI expression by MAIT cells across various inoculum: PBMC ratios for different treatment conditions as specified, with symbols representing individual donors and mean and SEM indicated by the bars. Statistical analysis comparing gE:gI expression by MAIT cells between different inoculum: PBMC ratios within each treatment condition was was performed via repeated measures one-way ANOVA (n = 3). *p<0.05, **p<0.01. (B) Flow cytometry plots depict co-expression of surface CD69 and intracellular IFN-γ for mock, exposed and infected MAIT cells in response to 5-OP-RU + IL-12/IL-18 treatment at the 1:20 inoculum: PBMC ratio. (C) Graphs show frequency of CD69 and IFN-γ co-expression of mock (blue), exposed (orange) and infected (red) MAIT cells to different treatment conditions for the various inoculum: PBMC ratios, with symbols representing individual donors and mean and SEM indicated by the bars. Statistical analysis comparing CD69 and IFN-γ co-expression expression of exposed and infected MAIT cells at different inoculum: PBMC ratios to mock control within each treatment group was performed via Two-Way repeated measures ANOVA (n = 3). *p<0.05, **p<0.01. (TIF) [file ppat.1012372.s004.tif]

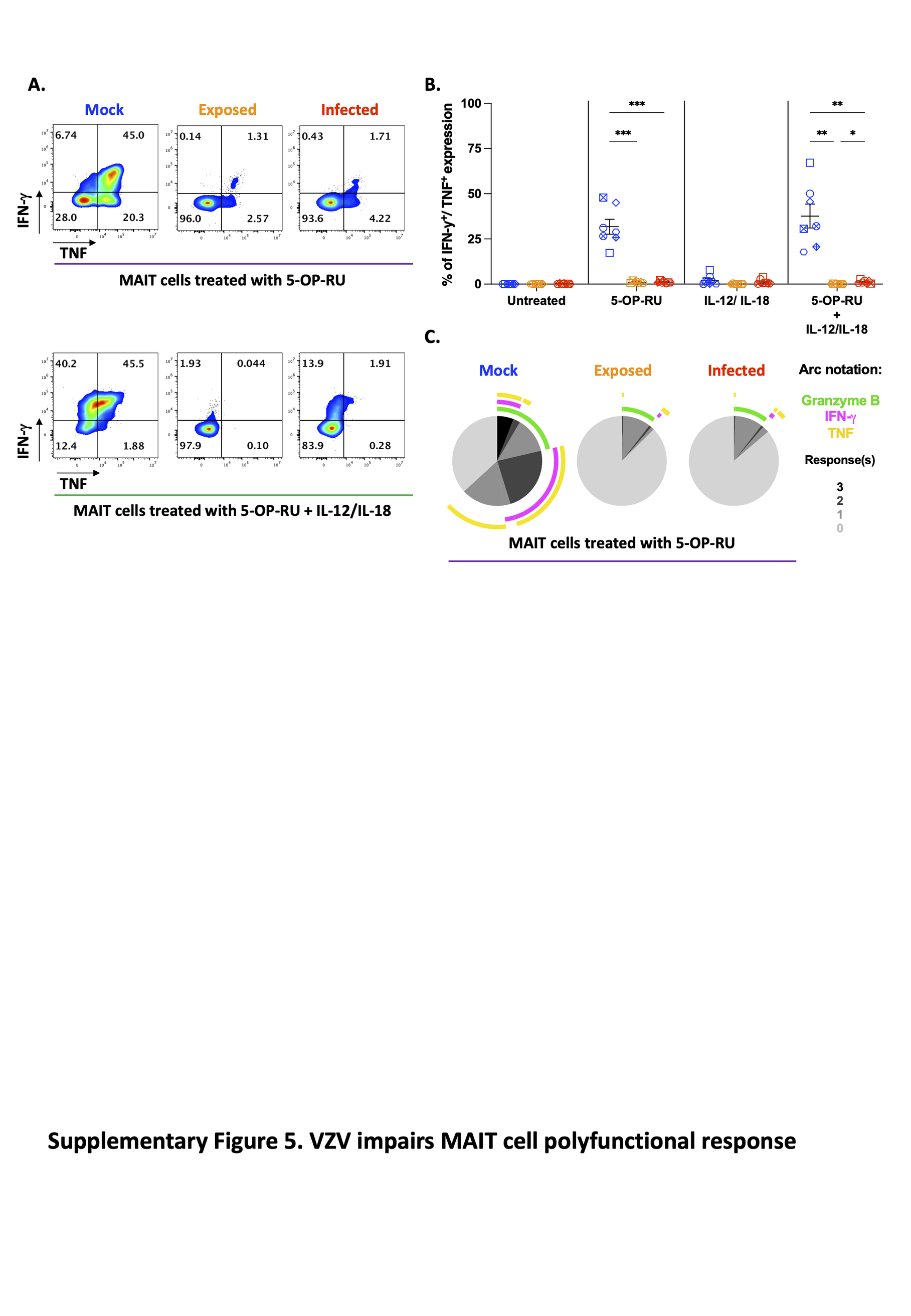

Supplement: S5 Fig — Human PBMCs were inoculated with mock or clinical VZV isolate (VZV-S) infected ARPE-19 epithelial cells for one day. PBMCs were removed from co-culture, before treating with different stimulations as specified, and then analysed by flow cytometry. (A) Flow cytometry plots depict intracellular co-expression of IFN-γ and TNF of mock, exposed and infected MAIT cells in response to 5-OP-RU and 5-OP-RU + IL-12/IL-18. (B) Graph show frequency of IFN-γ and TNF co-expression of mock (blue), exposed (orange) and infected (red) MAIT cells to treatments as specified with symbols representing individual donors and mean and SEM indicated by the bars. Statistical analysis comparing IFN-γ and TNF co-expression between mock, exposed and infected MAIT cells within each treatment group was performed via Two-Way repeated measures ANOVA (n = 7). *p<0.05, **p<0.01, ***p<0.001. (C) SPICE pie charts show the proportion of responses by mock, exposed and infected MAIT cells to 5-OP-RU stimulation, based on the combinations of granzyme B, IFN-γ and TNF expression. Pie slices indicate the number of responses (0–3) (key, bottom right). Arcs depict the markers detected for each response (key, top right). SPICE data represents the mean of seven donors. (TIF) [file ppat.1012372.s005.tif]
